# Genetic Diversity in Dutch Sheep Breeds

**DOI:** 10.1002/age.70088

**Published:** 2026-03-27

**Authors:** J. Noëlle Hoorneman, Mira A. Schoon, Richard P. M. A. Crooijmans, Sipke Joost Hiemstra, Jack J. Windig

**Affiliations:** ^1^ Wageningen University & Research Animal Breeding and Genomics Wageningen the Netherlands; ^2^ Centre for Genetic Resources the Netherlands Wageningen University & Research Wageningen the Netherlands

## Abstract

The Netherlands has a rich variety of native sheep breeds, most of them at risk or endangered. We studied the distinctiveness of these breeds, their genetic diversity within and between breeds, and how this diversity is shaped by geography, purpose, history and genetic management practices. Semen samples of 171 rams of 11 native Dutch breeds were genotyped with the IMAGE001 multispecies SNP chip including 10 K sheep SNPs. Genetic characterisation showed a clear genetic differentiation, except for the Texel and two Texel‐derived (sub)breeds. *F*_ST_ values were on average 0.124. The largest split was between breeds bred for milk or meat production on rich grasslands in the northwest of the Netherlands and breeds bred for grazing nature areas on poorer grounds in the east. The graphical distribution of the Principal Component Analysis mirrored the geographical distribution of the origin of the breeds. Within‐breed diversity was largest for the two breeds with largest population sizes (Texel and Kempen heath sheep) plus the one breed, Veluwe heath sheep, that consistently has used a breeding circle to maintain genetic diversity. The Flevolander, a more recently developed breed based on two foreign breeds, had the highest unique diversity of all breeds. The Texel received the highest ranking after the Flevolander when optimising contributions for the gene bank. As sudden changes in genetic diversity can occur also in large commercial breeds, we recommend that besides safeguarding the endangered breeds, cross sections of the Texel are also stored in the Dutch gene bank.

## 1 Introduction

Genetic diversity is the base for long term survival of populations of animals in nature and for maintaining viable livestock populations. It provides opportunities for breeders to select for desirable traits, and it enables populations to respond and adapt to changing environments. In breeds with small (effective) population sizes, inbreeding and genetic drift can increase rapidly and genetic diversity will be eroded (Wiener et al. 1992). Worldwide, at least 35% of the livestock breeds are at risk (FAO 2022), and production largely depends on just a few global high input/high output breeds.

Dutch sheep breeds form no exception, where the majority of the breed are endangered nowadays. The most dominant breed of Dutch origin is the Texel breed with over 20.000 purebred animals and the breed is also used a lot in crossbreeding. Historically, sheep have been farmed for the production of a wide range of products including meat, milk, wool, skins and manure. Different regions representing specific types of soil and environments harboured their own breeds. With the intensification of agriculture and the widespread use of artificial fertilisers, most breeds strongly decreased in numbers, except for the Texel breed. Genetic diversity has further been under pressure due to selection on productions traits, as well as for disease resistance (e.g., foot and mouth disease (Scudamore and Harris 2002), scrapie (Commission 2003; Windig et al. 2004) and bluetongue (Vellema 2008)).

The loss of genetic diversity within breeds can be mitigated with genetic management, such as breeding restrictions for rams, restrictions on inbreeding levels and rotational breeding schemes (Meuwissen 2009). An example of successful rotational breeding scheme in Dutch sheep breeds is the breeding circle that has been in use in the Veluwe heath sheep since the 1990s (Windig et al. 2019). Furthermore, gene banks have been set up in which genetic material (usually semen or embryos) is cryoconserved (Woelders et al. 2012). Gene banks serve as a backup in case of loss of genetic diversity due to, for example, an infectious disease outbreak, to support in situ populations when specific diversity or sire‐lines are lost in the live population and can serve as an archive for scientific research. Between 2001 and 2012, a cross section of nearly all Dutch sheep breeds (11 of 15 (sub)breeds) has been stored in the national Dutch Gene bank for livestock species (Hoving et al. 2013).

Genetic characterisation of gene bank material can contribute to optimisation of its use (Blackburn 2012; Doekes et al. 2018a; Zomerdijk et al. 2020). Compared to traditional pedigree information, genomic information can identify individual relationships within breeds more accurately and in more detail across the genome. Moreover, genomics enables us to accurately estimate relationships between breeds (Hanotte and Jianlin 2005; Toro et al. 2009). To genotype genetic collections at a low cost, a publicly available multispecies single nucleotide polymorphism (SNP) array, IMAGE001, was designed for the main livestock species with a focus on traditional breeds (Hiemstra et al. 2019; Crooijmans et al. 2020, 2025). We used this array to characterise genetic material of rams stored in the Dutch gene bank belonging to the native Dutch sheep breeds.

## 2 Breeds and Their History

**FIGURE 1:**
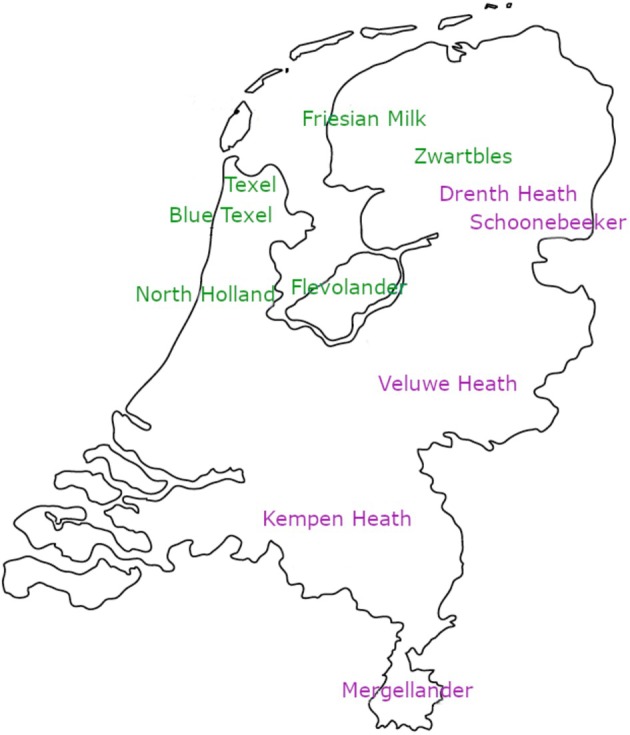
The geographic origin of the different Dutch native sheep breeds, where the pasture breeds (shown in green) are mostly in the northwest and the heath breeds (shown in purple) are spread more along the eastern side of the country from north to south.

There is a large variety in history, breeding goal, genetic management and geographic origin among the native Dutch sheep breeds. Historical relationships are largely anecdotical, making the origin of breeds and the amount of admixture uncertain. In general, two types of breeds can be distinguished: heath breeds and pasture breeds (similar to the British situation (Trow‐Smith 1957; Ryder 1964b)). The heath breeds are mainly found in the east of the Netherlands (Figure 1) on sandy soils with a lot of heath nature areas. Nowadays, these breeds are still used for conservation of nature reserves by grazing. The pasture breeds from north western parts (Figure 1), living on richer soils, are mainly used for the production of meat and milk. It is possible that different uses and differences in genetic management and selection pressure have resulted in differences in genetic diversity within and across the breeds.

Some of the breeds, in particular the heath sheep breeds, have ancient roots while others have been developed recently in the 70's. Archaeological finds dating from the Early Middle Ages uncovered likely ancestors of the Kempen heath sheep. Likewise, craniological research indicated that the Drenthe heath sheep is a direct descendant of the ‘turf’ (peat) sheep that were present in the Netherlands until the Middle Ages (Reitsma 1932). There are indications of introgression of Spanish Merino like sheep in the Schoonebeeker around 1600 and in the Kempen heath sheep in the early 1800s (Blink 1920; de Poel 2011).

Both the Texel and Friesian milk sheep originate from the white headed short tailed pasture sheep that were kept along the coast line from Denmark to North France. The current Texel was created by crossbreeding with English sheep breeds like the Leicester before 1900, followed by continuous selection for high meat production. Starting in 1968, breeders started breeding for a blue colour variant within the Texel breed. Even though there is a separate flockbook for the Blue Texel, there is regular exchange of breeding animals with the Texel flockbook. The Blue Texel can be considered as a subpopulation of the Texel breed. While the Texel breed continued to expand in numbers, the population sizes of the Friesian milk sheep dwindled, and this breed is now the smallest sheep breed in terms of (registered) population size in the Netherlands.

The most recently developed synthetic breeds, the Flevolander, North Holland and Swifter breeds, received the ‘Dutch native’ sheep breed status in the 2010s by passing the 40 year and 6 generation timeline (the definition of local or locally adapted breeds as per FAO (2013)). To improve the reproduction capacity of the Dutch sheep, the North Holland breed was created in 1975 by crossing Texel with Finnish landrace, and in the early 1970s, the Flevolander breed was created by crossbreeding Ile de France with Finnish land race sheep. The North Hollander breed remains to function like a crossbred population and still makes use of Texel rams for breeding. The Swifter is a breed developed from a similar cross in the 1970s—Texel × Flemish sheep—but this breed is not included in this study.

Apart from the North Holland and Blue Texel breed societies, which continue to accept some Texel ancestry, all these breeds have been bred in isolation from each other, at least since the start of the founding of their flockbooks, but most likely for much longer. There is some anecdotical evidence of the use of Kempen heath sheep in the Mergelland breed and the use of Schoonebeeker heath sheep in the establishment of the Zwartbles, although the latter was challenged by research on blood types (Buis and Tucker 1983).

All heath sheep suffered dramatic reductions in numbers in the early 1900s, and several were almost extinct around 1960 (van Helden and Minkema 1978; de Poel 2011). Breeding organisations and flockbooks were set up in the second half of the last century and numbers increased somewhat. Most successful in terms of number of animals is the Kempen heath sheep. All heath sheep breeds are now used in nature conservation for maintaining heath land by grazing with large flocks. The Veluwe heath sheep manages genetic diversity with the help of a breeding circle since the late 1970s (Windig et al. 2019), where rams are exchanged between flocks in a fixed rotational pattern. There are also other breeds that implemented a breeding circle, such as the Zwartbles, Flevolander and Friesian milk sheep, but those have been set up much more recently. Breeding restrictions for the rams (maximum number of ewes per ram) and maximum levels of inbreeding are used for the Drenthe and Schoonebeeker heath sheep.

In total, there are currently 13 Dutch breeds and 2 breeds that could be considered subbreeds. This study includes 1 subbreed (Blue Texel) and 10 breeds (Table 1). Nine of the evaluated breeds are endangered according to FAO risk status classification (FAO 2022) (FAO DAD‐IS/EFABIS (www.fao.org/dad‐is)). The number of registered breeding animals in the flockbooks in the Netherlands vary from < 300 for the Schoonebeeker heath sheep to 22 000 for the Texel sheep. For the Veluwe heath sheep due to the use of a breeding circle with multiple rams per flock, there is no parentage recording, and therefore, all breeding animals are registered in the supplementary section of the flockbook.

**TABLE 1:** Studied breeds, breed type, registered breeding males and females, effective population size (N_e_), population trend and risk level in 2022 (FAO 2022).

Transboundary breed name	Abbreviation	Type	Breeding M/F	N_e_	Population trend	Risk level	Founding flockbook
Zwartbles/Black blazed	ZB	Pasture	172/1584	61	Decreasing	At risk	1979
Blue Texel	BT	Pasture	507/4627	1250	Stable	At risk	1983
Texel	TX	Pasture	3289/22 020	—	Decreasing	Not at risk	1920s
North Hollander	NH	Pasture	90/2582	81	Increasing	At risk	1982
Flevoland	FL	Pasture	330/1775	151	Increasing	At risk	1971
Friesian milk	FM	Pasture	–/300	—	Decreasing	At risk	1908
Mergelland	MS	Heath	42/888	120	Decreasing	At risk	1978
Kempen heath sheep	KH	Heath	400/17 000	159	Increasing	Not at risk	1996
Veluwe heath sheep	VH	Heath	0/0	143	Stable	At risk	1999
Schoonebeeker heath sheep	SH	Heath	40/279	—	Stable	At risk	1991
Drenthe heath sheep	DH	Heath	179/1709	—	Stable	At risk	1985

Besides the number of breeding males and females, effective population sizes (N_e_) were estimated based on inbreeding rates for the breeds where this was available from pedigree data or simulations.

In this research, the genetic distinctiveness of the Dutch native sheep breeds is investigated. It shows the genetic relationships between the breeds and how they cluster according to geography, history and/or their use. Genetic diversities within and between breeds were calculated and studied in relationship with their use, population size and genetic management.

For optimal conservation management of these (mostly rare) breeds, the unique diversity of each breed is combined with genetic distinctiveness and genetic diversity to help in deciding which breed(s) should be prioritised for cryopreservation.

## 3 Materials and Methods

### 3.1 Genotyping

Genetic diversity in Dutch sheep breeds was evaluated by genotyping semen samples from gene bank collections of 10 Dutch native sheep breeds and supplemented with the genotypes of the Texel breed from France. The samples of the 10 Dutch native breeds were stored in the Dutch gene bank for animal genetic resources, managed by the Centre for Genetic Resources, the Netherlands (CGN) of Wageningen University & Research (Table 2). Because gene bank animals are selected to provide as good as possible a reflection of the whole population, gene bank collections are ideally suited to investigate genetic diversity in breeds. Pedigree information, mean kinships, different owners and different time periods are all used to ensure as much genetic diversity in the gene bank collection as possible. In addition, 21 Texel rams from France were added due to the lack of semen stored of the Dutch Texel breed in the Dutch gene bank.

**TABLE 2:** Dutch breeds used in this study, sample origins, sample sizes, mean callrate per breed and the number of unique alleles found in each breed.

Transboundary breed name	Dutch breed name	Sample origin	Number of sampled animals	Call rate	Unique alleles
Black blazed	Zwartbles	CGN genebank	19	0.994	5
Blue Texel	Blauwe Texelaar	CGN genebank	8	0.998	0
Texel	Texelaar	France	21	0.997	8
North Hollander	Noord Hollander	CGN genebank	5	0.993	1
Flevoland	Flevolander	CGN genebank	10	0.996	4
Friesian milk	Fries melkschaap	CGN genebank	17	0.994	4
Mergelland	Mergelland schaap	CGN genebank	18	0.995	3
Kempen heath sheep	Kempisch heideschaap	CGN genebank	18	0.997	1
Veluwe heath sheep	Veluws heideschaap	CGN genebank	18	0.995	5
Schoonebeeker heath sheep	Schoonebeeker heideschaap	CGN genebank	18	0.995	4
Drenthe heath sheep	Drents heideschaap	CGN genebank	19	0.995	12

Genotypes of 171 rams were included in this project. These rams were born between 1997 and 2014. Semen was collected between 2001 and 2015, both through ejaculate and epididymal semen collection. Each sample was genotyped with the IMAGE001v1 multispecies SNP array developed within the H2020 Image project (imageh2020.eu) (Hiemstra et al. 2019; Crooijmans et al. 2025).

### 3.2 Statistical Analyses

SNPs with a sample call rate < 95% were removed. The minor allele frequency threshold was set to 0.005, to avoid the loss of rare genetic variation. Only autosomal markers were used. There was no deviation from Hardy–Weinberg equilibrium detected.

Hierfstat and DartR were used to compute expected heterozygosity, observed heterozygosity and proportion of polymorphic alleles within breeds (Goudet 2005; Mijangos et al. 2022) using R statistical software v4.5.1 (R Core Team 2021).

### 3.3 Population Structure

To evaluate distinctiveness of the breeds and to estimate population stratification, a variational Bayesian framework in fastSTRUCTURE (Raj et al. 2014) was used. The software clustered the data according to allele frequencies into K populations (clusters). The predefined number of clusters (K) was initially set from 2 to 12 with a burnin period of 10 000 cycles and 20 000 data collection Markov chain Monte Carlo (MCMC) cycles. For each value of K, 3 runs were performed. The results of these runs were used to select the optimal number of clusters. The POPHELPER package (Francis 2017) in R calculated the change in likelihood between runs for each K, following the approach of Evanno et al. (2005). For the K's close to optimal, STRUCTURE was run again, but now with a burnin period of 100 000 cycles followed by 100 000 repetitions to confirm the detected patterns. To further evaluate the distinctiveness of the breed Neighbour Joining Trees were constructed. Genetic distances between rams were obtained as one minus the genetic similarity between them, with the similarity estimated from the genomic relationships (see below). From these distances NJ trees were generated using the Ape package in R (Paradis et al. 2004). The resultant trees were visualised using FigTree (Rambaut 2012). Breed relationships were visualised by plotting the PCA (Principal Component Analysis) scores of individuals for the first two principal components (PC). PCA's were performed by using the glPca command in the adegenet 2.1.11 package (Jombart 2008; Jombart and Ahmed 2011) and graphical representation was depicted using the ggplot2 4.0.1 package (Wickham 2016).

### 3.4 Genetic Diversity Between Breeds

Genetic diversity between breeds was evaluated by pairwise *F*_ST_ values.

Genomic kinships (G) between individuals were calculated with calc_grm version b202111 (Calus and Vandenplas 2013; Vandenplas et al. 2016), based on the count of identical alleles averaged across loci between two individuals (Nejati‐Javaremi et al. 1997; Eynard et al. 2015):

$$G_{\mathrm{jk}}=0.5+\frac{1}{2N}\left(\sum_{i}\left(x_{\mathrm{ij}}-1\right)\left(x_{\mathrm{ik}}-1\right)\right)$$

where *N* is the number of markers and *G*_*jk*_ is the estimated kinship between individual *j* and *k* across all markers, *x*_*ij*_ and *x*_*ik*_ are the genotype (0, 1 or 2 with 0 and 2 being the homozygous and 1 the heterozygous) of individual *j* and *k* for marker *i*. Note that other measures of genomic kinship exist (e.g., VanRaden 2008) where corrections are applied per marker for population allele frequencies. The measure we use is equivalent to using the formula described by VanRaden (2008) with the allele frequency set to 0.5 for each SNP. The average genomic kinship within breeds was used to evaluate genetic diversity. 1‐G was used as a measure of genetic diversity, following Frankham et al. (2002), since higher kinships indicate lower diversity.

Kinships were further analysed using the method of Eding et al. (2002). In this method, within‐breed and between‐breed genetic diversity are analysed simultaneously. A breed can have a high genetic diversity, but when this diversity is shared with another breed, it may not contribute much to the total genetic diversity of the whole set of breeds. On the other hand, a breed may have a low genetic diversity, but when this diversity is unique, it may still contribute significantly to the total genetic diversity. The total genetic diversity in the complete set of breeds based on kinships is given by

$$G_{\mathrm{div}}=1–c^{\prime }Fc$$

where **F** is the matrix of within‐ and between‐breed kinships, and c is a vector with the contribution of each breed, given as a fraction and thus summing up to 1. Optimal contributions are defined as the contributions that maximise the genetic diversity, that is, minimise the average kinship in the total set of breeds. Formula's to estimate optimal contributions are given in Eding et al. (2002). Breeds with the highest genetic diversity and least overlap with other breeds will receive the highest contributions. The unique genetic diversity of each breed can be quantified by calculating the difference between the total diversity in all breeds (the complete set) and the total diversity of the complete set minus the breed of interest.

To identify group‐specific SNPs, each SNP that passed the applied filtering criteria was analysed according to breed information. The breeds were grouped in pasture, heath and milk breeds. An allele was labelled as group‐specific if it was only present in one of the three groups and not detected in any of the other two, also called a private allele (Ramos et al. 2011).

## 4 Results

### 4.1 Genetic Diversity at Breed Level

From the 8558 SNPs on the array, 8272 remained after filtering and were used in the analysis. The number of polymorphic SNPs per breed is shown in Table 3.

**TABLE 3:** Diversity measures per breed. The number of loci (nLoc), observed heterozygosity (Ho), expected heterozygosity (He) and proportion of polymorphic loci.

	nLoc	Ho	He	Proportion polymorphic loci
ZB	7483	0.341	0.336	0.910
BT	8100	0.340	0.323	0.855
TX	7772	0.381	0.360	0.936
NH	8001	0.375	0.316	0.816
FL	7961	0.375	0.353	0.908
FM	7537	0.339	0.324	0.893
MS	7629	0.335	0.320	0.887
KH	7815	0.368	0.359	0.931
VH	7649	0.369	0.359	0.936
SH	7634	0.338	0.327	0.890
DH	7601	0.353	0.347	0.929
Mean		**0.356**	**0.338**	

The observed heterozygosity for all breeds was in line with the expected heterozygosity (Table 3). In all breeds the observed heterozygosity (Ho = 0.356) was slightly higher than the expected heterozygosity (He = 0.338) leading to a negative *F*_IS_ value close to zero (−0.0104).

The Texel breed had the highest observed heterozygosity (Ho = 0.381) whereas the Mergelland breed had the lowest (Ho = 0.335). The proportion of polymorphic loci overall was 0.978 and varied slightly between breeds, which was the highest in the Texel (0.936) and the lowest in the North Hollander (0.816).

### 4.2 Population Structure and Phylogenetic Analyses

**FIGURE 2:**
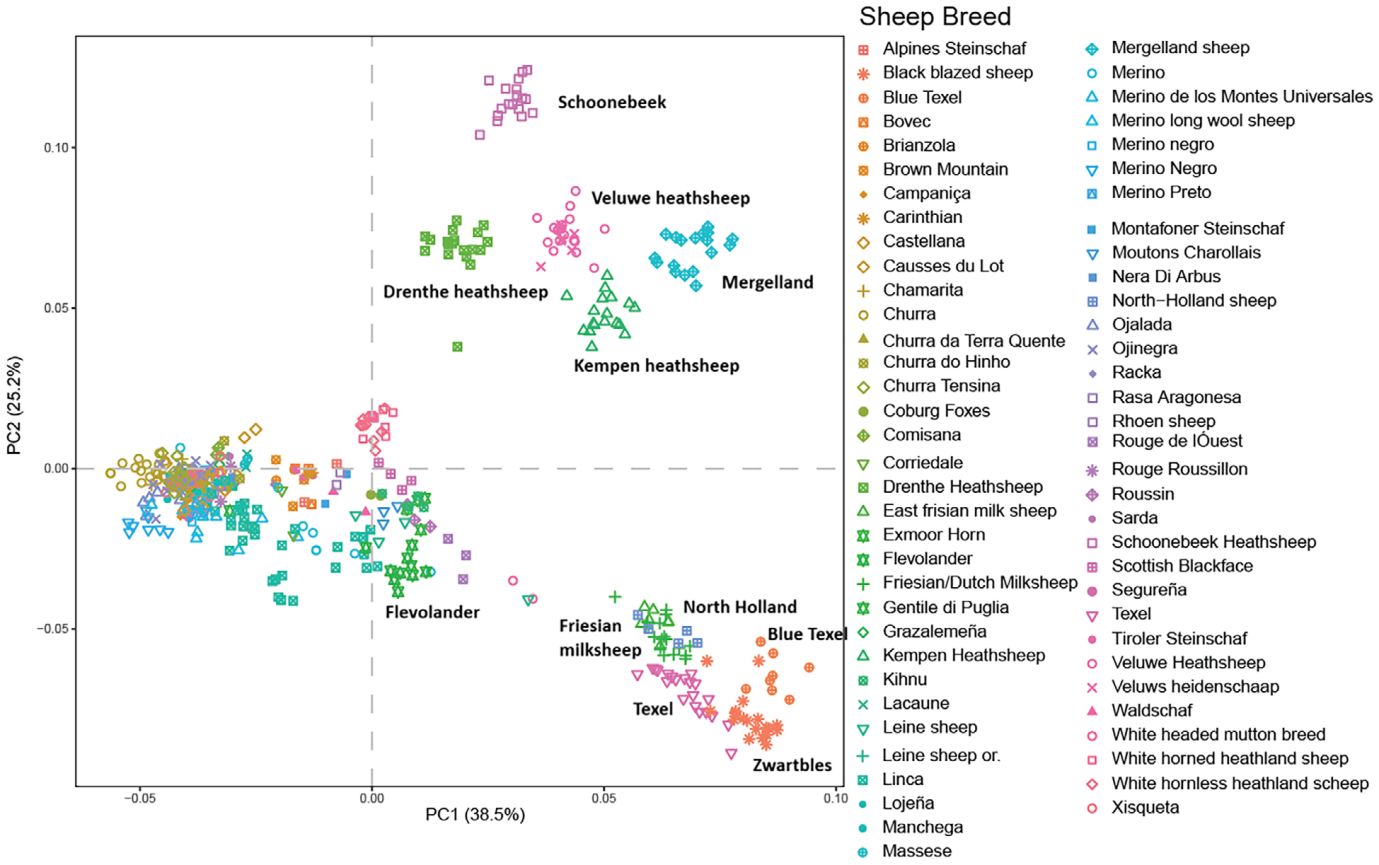
Principal component (PC) plot from 527 sheep from 66 breeds derived from 13 countries (Argentina, Austria, Estland, France, Germany, Hungary, Italy, The Netherlands, New Zealand, Portugal, Slovenia, Spain and the United Kingdom) from the paper of Crooijmans et al. 2025. The proportion of variance explained by each principal component is given in brackets. For this paper, the plot was edited by labelling the Dutch breeds for more clarity.

Our Dutch sheep breeds can be seen in a more international setting in this Principal Components plot (Figure 2) by Crooijmans et al. (2025). The Dutch breeds dominate almost half of the plot, spreading out along PC2 and a considerable part of PC1. A clear separation is seen between the heath breeds and the pasture breeds.

**FIGURE 3:**
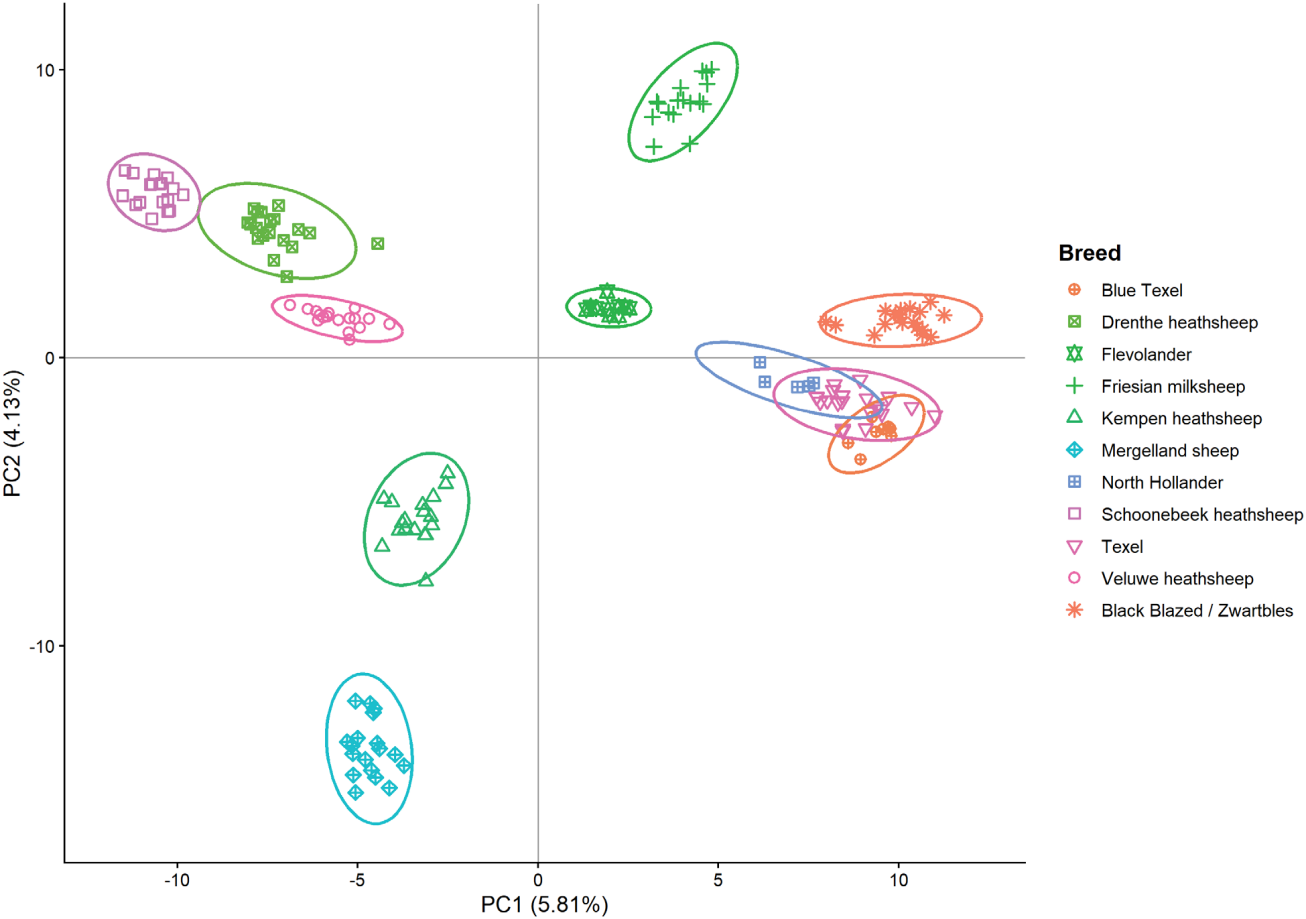
Principal Components Analysis for 8272 markers in the 11 breed populations. Every point is an individual ram; the ellipses are 95% confidence ellipses indicating the certainty about whether individuals truly belong in the specified group.

The PCA of only the Dutch breeds also showed a clear separation of the breeds as presented in Figure 3 with only minor overlap in the North Holland, Texel and Blue Texel breeds. The first PC explained 5.8% of the variation and the second PC 4.1%. Along PC1, the 5 heath breeds on the left are clearly separated from the 6 pasture breeds on the right. This mirrors the geographical distribution, as the heath breeds originated mostly east and the pasture breeds in the west. The only exception is the Zwartbles, which originated from the northeastern part of the Netherlands, but is shown on the right here along with the western pasture breeds. Along the 2nd PC, the breeds are geographically distributed from the north of the Netherlands (higher on PC2 axis) to the south (low on PC2 axis). Higher PCs did not provide much additional insight; PC3 highlighted the unique position of the Friesian milk sheep compared to the meat breeds and heath breeds. Plots of PC 1 & 3 and PC 2 & 3 can be found in Figure S1.

**FIGURE 4:**
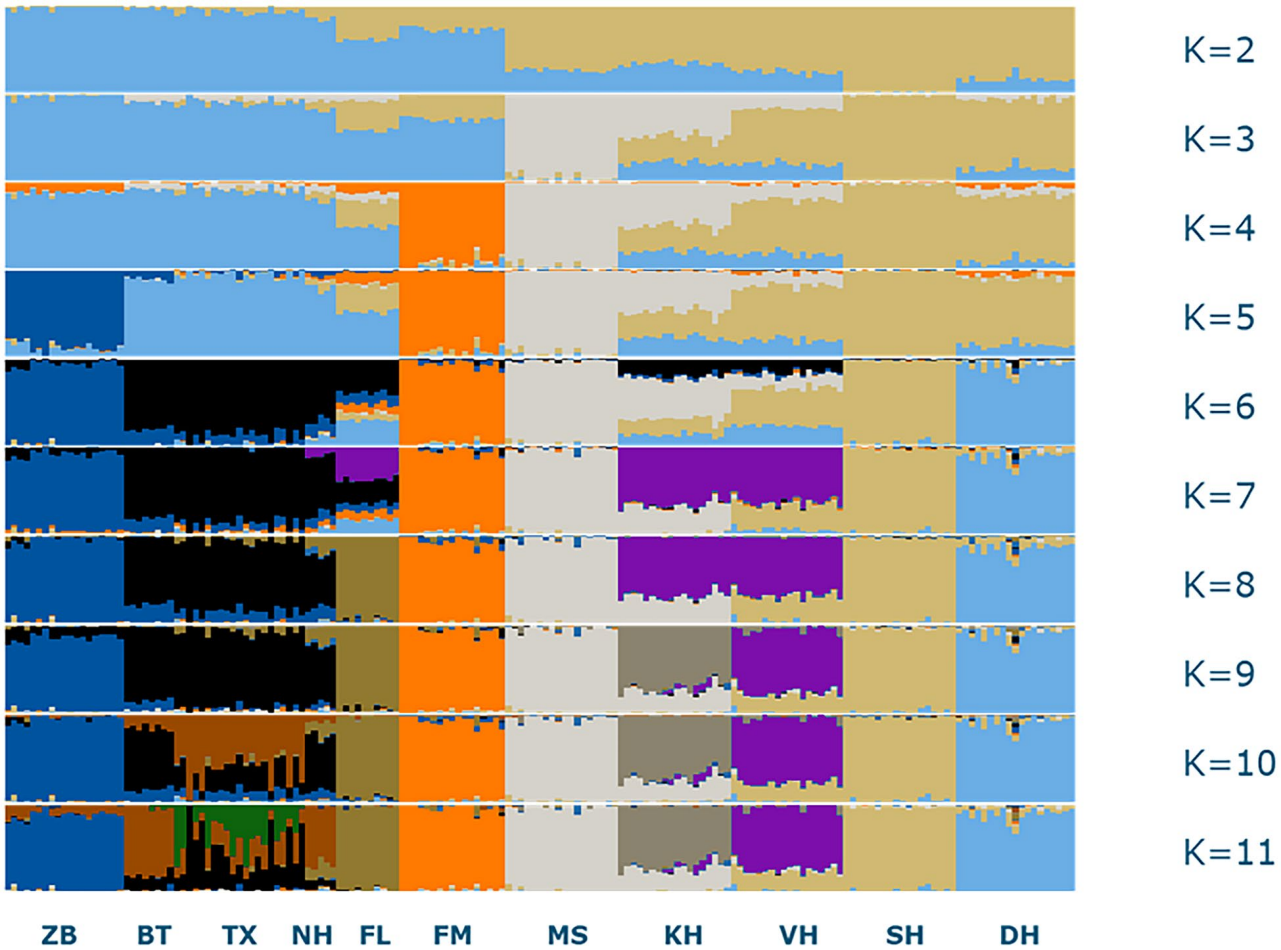
Summary of plots of estimate of Q for *K* = 2–11 in 11 Dutch sheep populations. Each individual is represented by a single vertical column divided in one or more coloured segments. Each colour represents a suggested cluster (K). BT, Blue Texel; DH, Drenthe heath sheep; FL, Flevolander; FM, Friesian milk sheep; KH, Kempen heath sheep; MS, Mergelland sheep; NH, North Hollander; SH, Schoonebeeker heath sheep; TX, Texel; VH, Veluwe heath sheep; ZB, Zwartbles/Black blazed sheep.

The optimal number of clusters in the STRUCTURE analysis was 10. With K = 2 heath breeds were separated from the pasture breeds (Figure 4). With each additional cluster a single ‘new’ breed was detected up to *K* = 10. This started with the Mergelland sheep (*K* = 3), followed by the Friesian milk sheep (*K* = 4) and the Zwartbles separated from the other pasture breeds (*K* = 5). *K* = 6 detected a cluster for the Drenthe heath sheep. Adding a seventh cluster separated the Veluwe heath sheep and Kempen heath sheep from the Schoonebeeker. *K* = 8 added a cluster for the Flevolander and *K* = 9 placed the Kempen heath sheep in its own cluster. Clusters within the group Blue Texel, Texel and North Holland breed were made with *K* = 10 and *K* = 11, but these breeds showed considerable overlap especially between the North Holland and the Blue Texel.

**FIGURE 5:**
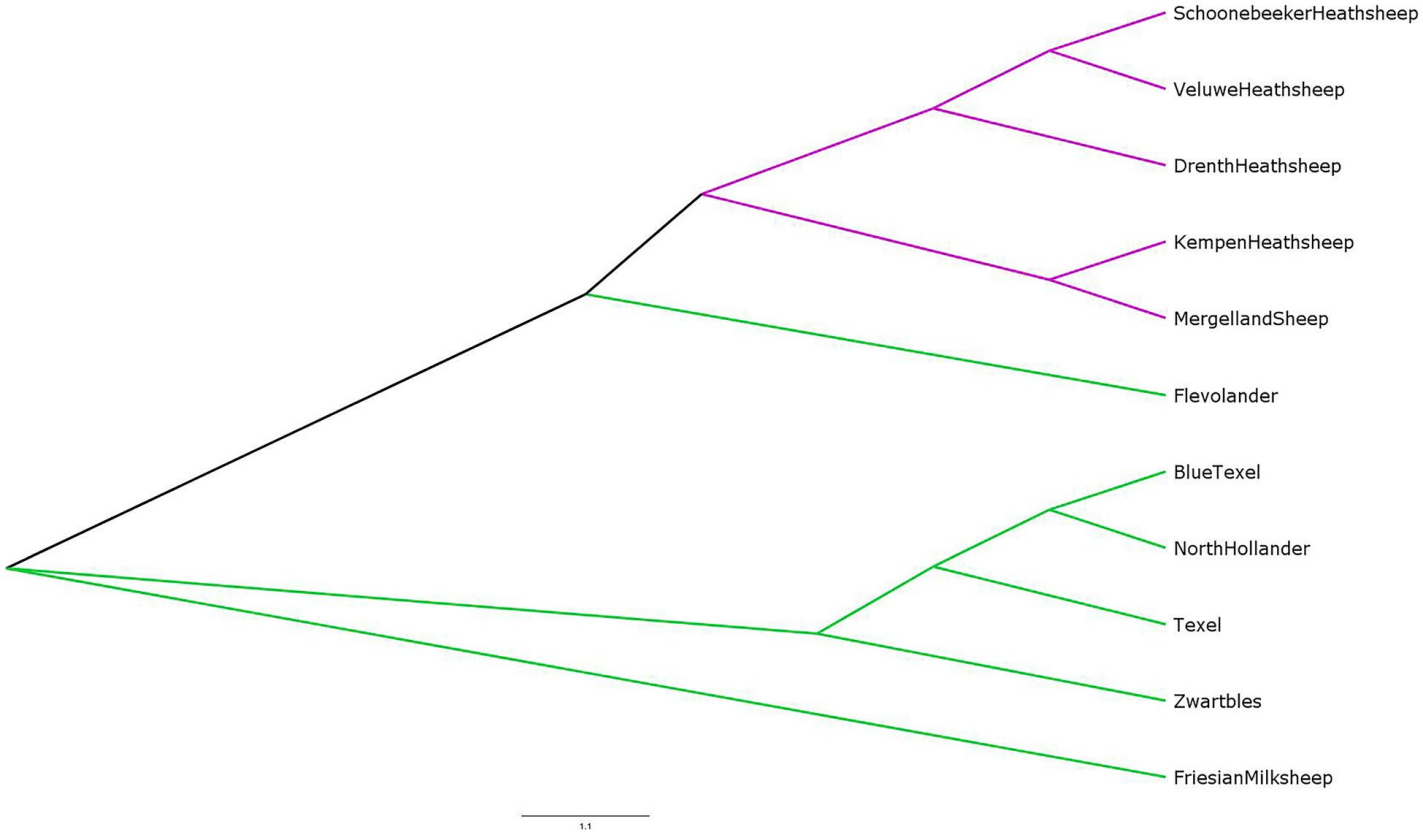
The phylogenetic tree depicts the lines of evolutionary descent of the different sheep breeds. Purple lines depict the heath breeds, green lines depict the pasture breeds.

The Neighbour Joining Tree shows very distinct clustering of the different breeds (Figure 5). Similar to the results of the PCA and STRUCTURE, there was a clear distinction between the heath breeds and the pasture breeds. However, the Flevolander and the Friesian milk sheep are not branched within those two types. The Flevolander is placed closer to the heath breeds but splits off far before any of the other breeds. The Friesian milk sheep is separated from both the heath breed cluster and the pasture breed cluster.

The Neighbour Joining Tree of all individual sheep shows a clear classification of all individuals within their own breed accordingly (Figure S2). Even for the breeds much more closely related, such as the Blue Texel (sub population of Texel) and the North Hollander (synthetic breed with Texel as one of the founders), all sheep were assigned to the correct group.

One Kempen sheep got sorted into a different group with no other animals or breeds and the branch was on the same height as the other Kempen sheep.

### 4.3 Genetic Diversity Between Breeds

Genetic differentiation among breeds was moderate to high (mean *F*_ST_ = 0.124, range = 0.061–0.186), indicating clear breed structure (Table S1). In contrast, within‐breed inbreeding coefficients were close to zero for all breeds, suggesting that populations are approximately in Hardy–Weinberg equilibrium with little evidence for heterozygote deficit. The smallest distances, lowest *F*_ST_ values, were found between the Kempen and Veluwe heath sheep (*F*_ST_ = 0.070) and between the Texel, Blue Texel and the North Hollander (0.061–0.079). The highest *F*_ST_ values, the largest distance between breeds, were found for the Friesian milk sheep and Mergelland (0.187) and between the Blue Texel and the Schoonebeeker heath sheep (0.186), which is in line with the PCA results.

The highest average kinships, the lowest genetic diversity within the breed, were found in the Blue Texel (*F* = 0.77), North Hollander (*F* = 0.81) and Mergelland sheep (*F* = 0.74) (Table 4). The lowest average kinship in this dataset, the breeds with the highest genetic diversity, were found in the largest population, the Texel breed (*F* = 0.59). Second lowest kinships were found for the Veluwe heath sheep and the second largest breed, Kempen heath sheep (*F* = 0.60).

**TABLE 4:** The average genomic kinships within and between breeds. The within‐breed kinships are shown on the diagonal, the between breed kinships below and above the diagonal. The colours correspond to the amount of kinship, with green for the lowest mean kinships and red for the highest.

	ZB	BT	TX	NH	FL	FM	MS	KH	VH	SH	DH
**ZB**	0.6821	0.4414	0.4116	0.4086	0.3421	0.3925	0.3668	0.3600	0.3552	0.3422	0.3304
**BT**	0.4414	0.7747	0.4782	0.5088	0.3371	0.3658	0.3844	0.3723	0.3588	0.3304	0.3272
**TX**	0.4116	0.4782	0.5884	0.4325	0.3365	0.3548	0.3595	0.3555	0.3427	0.3239	0.3219
**NH**	0.4086	0.5088	0.4325	0.8142	0.3520	0.3644	0.3645	0.357	0.3577	0.335	0.3358
**FL**	0.3421	0.3371	0.3365	0.3520	0.6446	0.3298	0.3266	0.3261	0.3305	0.3242	0.3271
**FM**	0.3925	0.3658	0.3548	0.3644	0.3298	0.7278	0.3667	0.3548	0.3604	0.3568	0.3523
**MS**	0.3668	0.3844	0.3595	0.3645	0.3266	0.3667	0.7442	0.4629	0.403	0.3945	0.3792
**KH**	0.3600	0.3723	0.3555	0.357	0.3261	0.3548	0.4629	0.5966	0.4086	0.3939	0.3740
**VH**	0.3552	0.3588	0.3427	0.3577	0.3305	0.3604	0.403	0.4086	0.5961	0.4495	0.4012
**SH**	0.3422	0.3304	0.3239	0.335	0.3242	0.3568	0.3945	0.3939	0.4495	0.7188	0.4187
**DH**	0.3304	0.3272	0.3219	0.3358	0.3271	0.3523	0.3792	0.3740	0.4012	0.4187	0.6374

In Table 4 the pasture breeds are listed in green followed by the heath breeds in purple. Within those groups, the between breed kinships are relatively high, and between the groups, the between‐breed kinships are much lower. The most notable breed is the Flevolander with all between breed kinships below 0.36. The two northern heath breeds Schoonebeeker heath sheep and Drenthe heath sheep have the lowest between‐breed kinships with the pasture breeds.

The contribution of the breeds that maximise the genetic diversity of the whole set of breeds was variable across breeds. The largest contribution was for the Flevolander (Table 5), which combines a relatively high within breed genetic diversity with low kinships (Table 4) with all other breeds. Next in line was one pasture breed and one heath breed. The Texel had the highest within genetic diversity of all breeds and the second highest unique diversity. The Drenthe heath sheep had equally high unique diversity as the Texel, and although its within‐breed genetic diversity was somewhat lower than some of the other heath breeds, it did receive the highest contribution of all heath breeds. On the other end of the spectrum is the Blue Texel with the lowest contribution and least unique diversity. This breed combines a low within‐breed diversity with a large overlap with the Texel and the North Holland.

**TABLE 5:** The genetic diversity (= 1—average genomic kinship) within the breed, the optimal contribution for each breed in order to minimise the average kinship of the whole set of breeds and unique diversity of each breed.

Breed	G_div_	Contributions	Unique diversity
Zwartbles	0.318	7.4%	0.0017
Blue Texel	0.226	1.8%	0.0001
Texel	0.412	15.5%	0.0052
North Holland	0.186	4.1%	0.0007
Flevolander	0.356	18.5%	0.0131
Friesian milk sheep	0.273	9.5%	0.0037
Mergelland Sheep	0.256	3.9%	0.0005
Kempen heath sheep	0.404	10.5%	0.0024
Veluwe heath sheep	0.404	8.5%	0.0015
Schoonebeeker heath sheep	0.282	7.0%	0.0016
Drenthe heath sheep	0.363	13.2%	0.0052

**FIGURE 6:**
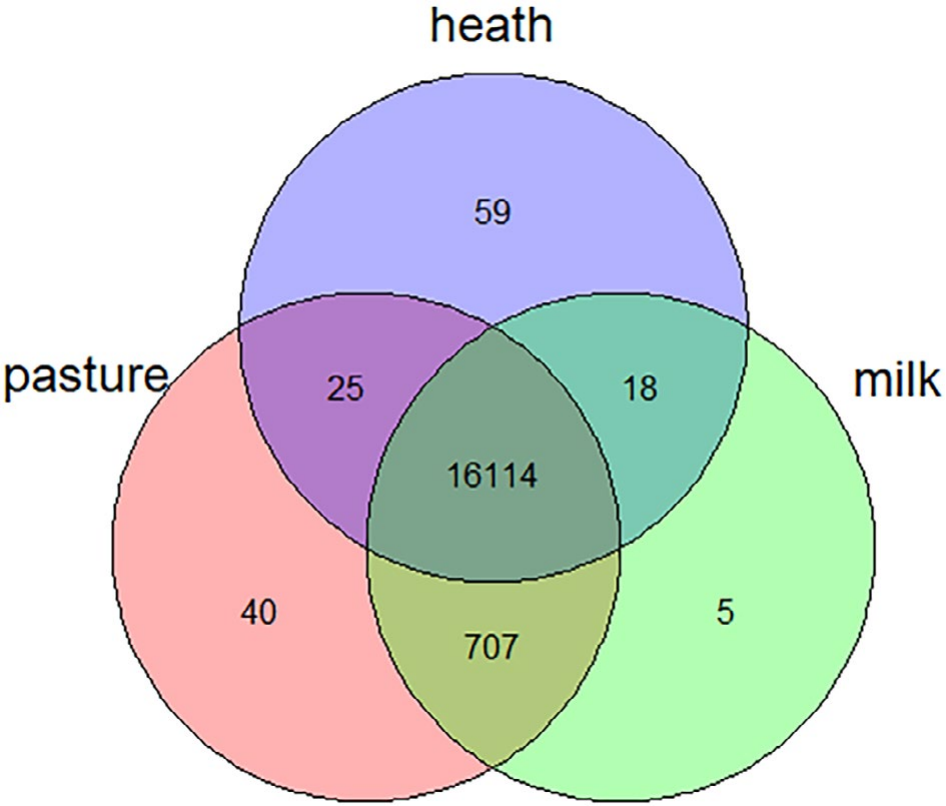
Venn diagram showing the number of shared and group‐specific variants in each group; heath breeds (Mergelland, Kempen, Veluwe, Schoonebeeker, Drenth), pasture breeds minus the milk breed (Zwartbles, Blue Texel, Texel, North Holland, Flevolander) and the milk breed (Friesian Milk).

Among the set of 8272 SNPs, 0.6% were identified as putatively group‐specific (Figure 6), indicating that the genotype of one of the alleles was present in only one of the three groups (pasture, heath and milk). SNPs specific for heath breeds were the most abundant (59), while the milk breed displayed the lowest number of group‐specific SNPs (5). The overlap between the milk breed and the other pasture breeds was much larger (707) than between the heath breeds and either the milk (18) or pasture (25) breeds. Over 16,114 of the variants occurred in all three groups.

Unique alleles were also found in all breeds, except for the Blue Texel (Table 2). The Drenth heath sheep had the most unique alleles (12), and the North Hollander and Kempen heath sheep both only had 1 unique allele.

## 5 Discussion

### 5.1 Distinctiveness

We characterised 11 breeds, with average *F*_ST_ values between breeds of 0.124, which is higher than the average *F*_ST_ of 0.061 reported for Spanish breeds (Álvarez et al. 2004) and 0.107 in the Welsh breeds (Beynon et al. 2015). On a worldwide scale, our results fell in the middle of the reported range of 0.053–0.25 (Kijas et al. 2009; Hall 2022). The highest F_ST_ values found within our study were between the heath and pasture breeds.

The split between heath and pasture breed was most prominent in all analyses. The clear genetic separation between the pasture and heath breeds coincides with both a geographic separation (west/north vs. east/south) separating regions with poor, sandy soils from more humid regions with richer peat or clay soils. Breeds in the regions have a different use as well: meat production vs. grazing nature conservancy areas. Consequently, the pasture breeds have been selected for high production rates for meat or dairy on richer soils and diets, while the heath breeds have been selected to thrive on poorer soils and feed quality and to be able to cover larger distances. In a national survey of Dutch cattle farms on herd characteristics a similar split between West/North and South/East was evident, with two native cattle breeds originating from the West/North and one from the South/East (Bonekamp et al. 2025). In British sheep breeds, a similar distinction is made between heath and pasture breeds matching historical observations (Trow‐Smith 1957; Ryder 1964a).

When comparing the breeds within the heath and pasture group a distribution of breeds based on geography was observed in the PCA analysis. The PCA mirrored geographic origin of the landrace breeds, with a continuous north to south cline when ranked on PC2. Position of synthetic breeds such as the North Hollander and Flevolander were more likely based on the high relatedness to the Texel in case of the North Hollander and genetic distance to the other breeds in case of the Flevolander. Breeds which are geographical close showed the lowest *F*_ST_ values and highest average relatedness such as between Texel and Texel‐derived breeds, and between the geographically adjacent Schoonebeeker and Veluwe heath sheep. On the other hand, within the heath sheep the Schoonebeeker and Mergelland sheep, which are geographically the widest apart had the highest *F*_ST_ values and lowest average relatedness. Geographic clustering matched by genetic relatedness has been observed in several countries, for example, in Italy (Ciani et al. 2014), Ethiopia (Edea et al. 2017), Croatia (Drzaic et al. 2022) and Sweden (Rochus et al. 2020), as well on a European scale (Lawson Handley et al. 2007) and a worldwide scale (Kijas et al. 2012). Migration on a local scale and associated gene flow apparently strongly influences patterns of genetic relations between breeds across the world.

The Zwartbles was the exception as it did not cluster genetically with the Schoonebeeker and Drenthe heath sheep, which are geographically the closest. Indeed, breed organisations and certain breeders of the Zwartbles sheep called their sheep ‘improved Schoonebeekers’ and claimed that purposeful selection in their Schoonebeeker sheep had led to the development of the Zwartbles. We show, however, that the Zwartbles is much more closely related to the Texel breeds, and its *F*_ST_ value with the Schoonebeeker is one of the highest. This confirms results from one of the earliest researches on DNA markers in the Netherlands (Buis and Tucker 1983) that showed that blood groups of Zwartbles resembled those of Texel sheep and not those of the Schoonebeeker. It is clear that if there has been any genetic influence of the Schoonebeeker it must have been small and selection must have operated against it. The Zwartbles was always selected for commercial characteristics such as fertility, milk production and meat, and not for traits that are important for grazing nature areas.

Genetic diversity of breeds is not fixed and changes constantly over time. A relatively recent addition to the Dutch sheep breeds is the Flevolander. The Flevolander originated from Finnish Landrace animals crossed with Ile de France in the 1970s. This explains the low genetic similarities between the Flevolander and all other Dutch breeds. Two other recent additions are the North Hollander and the Blue Texel. Both breeds have until recently approved Texel ancestries and cluster still close to their ancestral breed. The North Hollander originated from a cross between Finnish Landrace and Texel sheep, so a closer relatedness with the Flevolander was expected. Apparently, continuous crossing of the North Hollander with Texel sheep has diminished its relatedness with the Flevolander. Based on the results of this study, the distinctiveness of the North Hollander and Blue Texel could be disputed. However, both the North Hollander and Blue Texel may continue to develop in a different direction than the Texel breed, that is, by selection and/or genetic drift, and monitoring genetic diversity in the future remains important.

Apart from the Texel breeds, all other breeds are clearly genetically distinctive. In the individual phylogenetic, 170 of the 171 animals were correctly assigned to their breed, even in the closely related breeds such as the Texel and Blue Texel. The only individual that was not correctly assigned to its breed is a Kempen ram from 1999. This ram was from a separated small flock from a different region of which it was the only animal sampled. In the structure analyses, though, it was correctly assigned to the cluster with Kempen animals.

### 5.2 Diversity

The genetically most diverse breeds were the Texel, Kempen heath sheep and Veluwe heath sheep according to all measures of genetic diversity within breeds, while the Mergelander, Blue Texel and North Hollander showed lowest diversity. The relatedness within breeds (Table 4) was with 0.974 strongly correlated with the expected heterozygosity (Table 3). The expected heterozygosity ranged from 0.32 to 0.38 (mean 0.34), which is at the high side of the range of 0.26–0.37 (mean 0.32) found across Northern European breeds (Kijas et al. 2012). Although all the Dutch breeds with lower genetic diversity can be considered at risk based on their population size, they are not as endangered as some other Northern European breeds. The lower diversity of the Blue Texel and the North Hollander may in part be caused by the lower number of samples. The Texel and the Kempen heath sheep have the highest population sizes and, together with the Veluwe heath sheep, the highest genetic diversity. On the other side, the Mergelland sheep has one of the smallest population sizes of the Dutch breeds, which may explain its lower genetic diversity. It does, however, have a part of the breed living in Belgium and a lot of smallholders contributing to maintaining levels of genetic diversity (Windig et al. 2007). The Mergelland and Kempen sheep are both transboundary breeds that also have an active breeding population in Belgium. These populations both have low genomic LD‐based N_e_ estimates (resp. 21 and 18) and a strongly skewed sex ratio around 3% which highlights their vulnerability (Meyermans et al. 2024). Due to the restrictions of the size of the SNP array used for this study, no reliable genomic N_e_′s can be estimated to compare to the Belgian populations. Future research including a larger SNP array and larger sample sizes could be interesting to fill this gap, or alternatively gene bank data on sheep born in different time periods could be used to calculate inbreeding rates and N_e_.

The Veluwe heath sheep is a small and local breed and has a population size comparable to or smaller than most other Dutch sheep breeds. Its high genetic diversity is thus somewhat unexpected, but may be explained by the genetic management applied. It has consistently used a breeding circle since the late 1970s to mitigate inbreeding rates. DNA analysis confirmed that the effective population size was relatively large for such a small local breed with no more than 10 flocks, N_e_ = 161 based on 0.31% inbreeding rate (Windig et al. 2019). The breed next in line with regard to genetic diversity is the third largest breed, the Flevolander. It also uses, for a part of the population, a breeding circle to manage inbreeding levels with rams from 10 different bloodlines.

In conclusion, the genetic diversity seems strongly influenced by population size and genetic management.

### 5.3 Unique Diversity

There is clear indication of different genomic make‐ups of each breed and this might open the door to the use of unique SNPs for breed assignment (Hulsegge et al. 2019). There were small numbers of group‐specific SNPs, indicating that each has some unique genetic identity although the percentages were low. Also, each breed had unique alleles that were not present in any of the other breeds; only the subbreed Blue Texel did not. Because the sample sizes for some breeds in this study were very low (*n* = 5 for the North Hollander) and other breeds were not represented at all, present results are not sufficient to develop a breed assignment test for all Dutch sheep breeds. This study used 8272 SNPs and while this could be enough, more breed‐specific alleles could be detected when using a larger SNP array. Further research with all Dutch breeds, a larger sample size and/or more markers would be interesting to confirm the presence of enough unique diversity and potential for developing an accurate breed assignment test.

### 5.4 Lessons for Conservation

All breeds except for the cluster of Texel, Blue Texel and North Hollander were genetically clearly distinct, and thus it is valuable to conserve them in the gene bank. For both the Blue Texel and North Hollander, animals with a parent from the Texel flockbook are still eligible for registration, which could explain the observed genetic overlap. In the Neighbour joining tree both the Blue Texel and North Hollander were clustered as a single unit, though, so some distinctiveness, albeit small, is present. Nevertheless, even though breeds are not (yet) fully distinct, it can be valuable to conserve genetic diversity that lies at the foundation of a breed. This is especially true if the ancestral breed is not conserved in the gene bank, as is the case with the Texel.

Genetic distinctiveness is not the only criterion to prioritise breeds for conservation in the gene bank. Genetic diversity within a breed is an important aspect as well. Taking both within‐ and between‐breed diversity into account, the Flevolander receives the highest contributions when optimising contributions for the gene bank and it has the highest unique diversity of all breeds (Table 5). The Flevolander originates from a cross between the two foreign breeds Finnish Landrace and Ile de France, causing low genetic similarities between the Flevolander and the other Dutch breeds. Probably, it will be less unique from a European perspective if both ancestral breeds are taken into account. Nevertheless, since it has developed now for over 50 years independently from its ancestral breeds some unique diversity even at a European scale cannot be ruled out.

The Texel, which has the highest amount of within‐breed genetic diversity of all analysed breeds was the next in line for prioritisation, when taking both within‐ and across‐breed diversity into account. Its high genetic diversity is probably a consequence of its large population size, both on a national scale and worldwide. At the same time this is also the reason why it has not been stored in the gene bank because priority has been given to breeds at risk. This is also true for the Swifter and the Dutch Spotted sheep, two breeds that were not included in this study as they weren't present in the gene bank. However, it is important to store genetic diversity of the large commercial breeds, that dominate food production, in gene banks as well. For the Holstein cattle breed, dominating dairy production worldwide, sudden changes in genetic diversity have occurred (Doekes et al. 2018b), and it is important to conserve lost diversity, in case it may be needed in the future. The large collection of cryoconserved genetic material for the Holstein proved to be of high value in case breeding goals will change (Doekes et al. 2018a). Moreover, gene banks can serve as a backup in case disasters occur in the breeding programs for commercial companies, reason why Holstein material is stored in the Dutch gene bank. Therefore, we recommend that cross sections of the Dutch Texel and the Swifter are stored in the Dutch gene bank as well.

## 6 Conclusion

The Dutch native sheep population consists of a number of genetically distinct breeds. All genetic analyses performed clearly differentiated sheep breeds according to their historical origins and geographical distribution. Both the Dutch gene bank and the IMAGE multispecies array proved their value in enabling a comprehensive analysis of the genetic diversity of Dutch sheep breeds without the lengthy process of sampling genotypes across breeds in flocks scattered across the country.

This study not only provided more insight in the structure of the Dutch native sheep population, but it proved valuable for assessing the effects of selection and genetic management measures. Next to breeds with a large population size, a breed with genetic management in the form of a breeding circle had a high genetic diversity as well. Additionally, insight in genetic diversity of the Dutch gene bank collection, in contrast to the in situ population, can support identification of unique males in the live population to be added to the gene bank collection.

## Conflicts of Interest

The authors declare that the research was conducted in the absence of any commercial or financial relationships that could be construed as a potential Conflicts of Interest.

## Supporting information


**Figure S1:** Principal components analysis for 8272 markers in the 11 breed populations. Every point is an individual ram, the ellipses are 95% confidence ellipses indicating the certainty about whether individuals truly belong in the specified group.
**Figure S2:** The Neighbour Joining Tree of all individual sheep. Different breeds are indicated with different colours. Dark purple is Mergelland, orange is Kempen heath sheep, light purple is Schoonebeeker heath sheep, pink is Veluwe heath sheep, lime green is North Hollander, red is Blue Texel, light blue is Texel, brown is Zwartbles, yellow is Flevolander, blue is Friesian milk sheep, and green is Drenthe heath sheep.
**Table S1:** *F*_ST_ values between breeds and *F*_IS_ values within breeds.

## Data Availability

The data that support the findings of this study are openly available in European Variation Archive at https://www.ebi.ac.uk/ena/browser/view/PRJEB57696, Project: PRJEB57696.
