# Supplementary material for: Genetic Diversity in Dutch Sheep Breeds
Source: Anim Genet. 2026 Mar 27;57(2):e70088. doi: 10.1002/age.70088 (PMC13025069; doi:10.1002/age.70088)
Supplement: Supplementary file 1 — Figure S1: Principal components analysis for 8272 markers in the 11 breed populations. Every point is an individual ram, the ellipses are 95% confidence ellipses indicating the certainty about whether individuals truly belong in the specified group. Figure S2: The Neighbour Joining Tree of all individual sheep. Different breeds are indicated with different colours. Dark purple is Mergelland, orange is Kempen heath sheep, light purple is Schoonebeeker heath sheep, pink is Veluwe heath sheep, lime green is North Hollander, red is Blue Texel, light blue is Texel, brown is Zwartbles, yellow is Flevolander, blue is Friesian milk sheep, and green is Drenthe heath sheep. Table S1: F ST values between breeds and F IS values within breeds. [file AGE-57-0-s001.docx]

# Supplemental Material

*Supplemental* *Figure 1: Principal Components Analysis for 8,272 markers in the 11 breed populations. Every point is an individual ram, the ellipses are 95% confidence ellipses indicating the certainty about whether individuals truly belong in the specified group.*

*
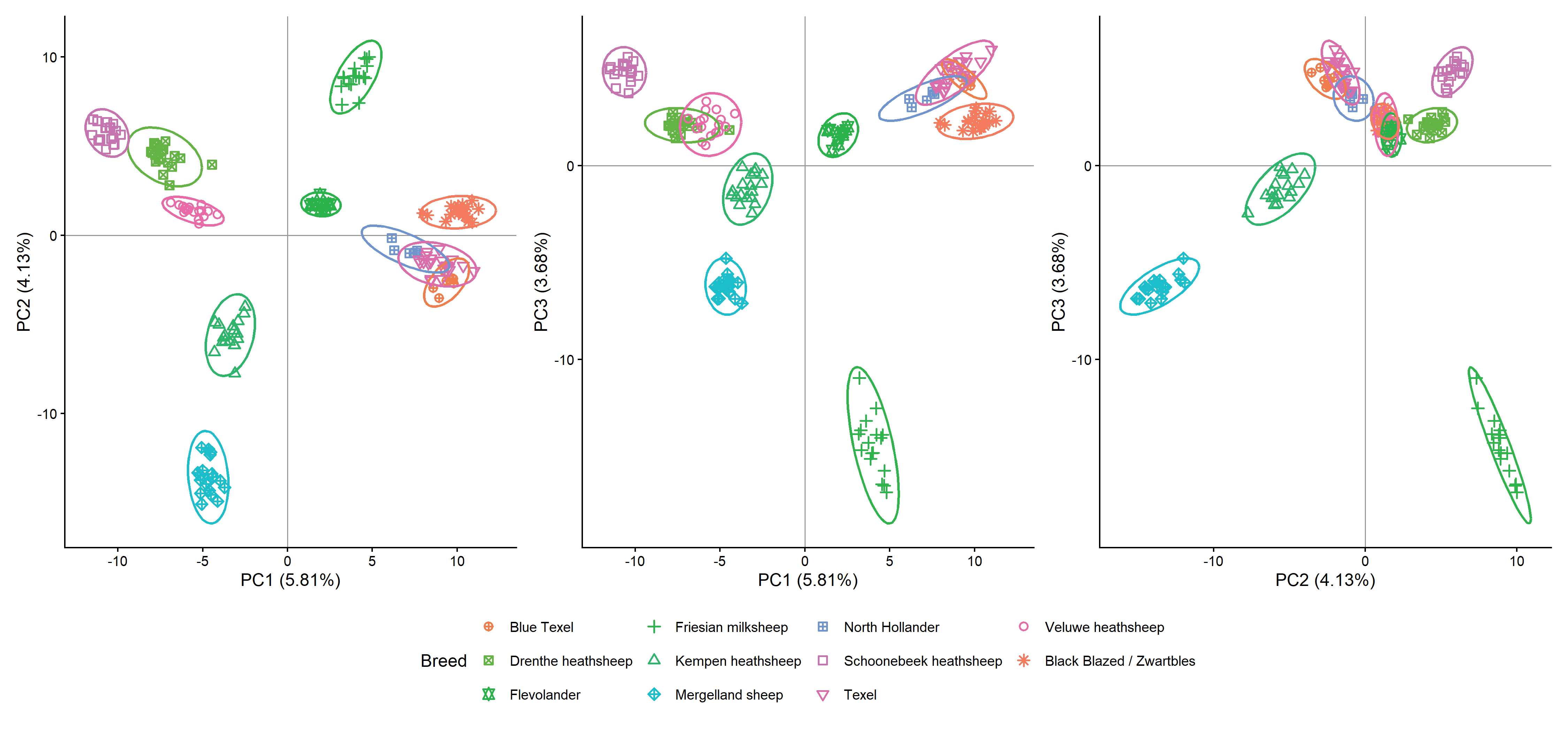
*

*Supplemental Table 1: F_ST_ values between breeds, and F_IS_ values within breeds.*

|  | **ZB** | **BT** | **TX** | **NH** | **FL** | **FM** | **MS** | **KH** | **VH** | **SH** | **DH** |
| --- | --- | --- | --- | --- | --- | --- | --- | --- | --- | --- | --- |
| **ZB** | 0.010 | 0.113 | 0.100 | 0.125 | 0.136 | 0.153 | 0.173 | 0.127 | 0.130 | 0.177 | 0.158 |
| **BT** | 0.113 | 0.005 | 0.061 | 0.067 | 0.138 | 0.170 | 0.168 | 0.120 | 0.127 | 0.186 | 0.159 |
| **TX** | 0.100 | 0.061 | -0.033 | 0.079 | 0.110 | 0.144 | 0.148 | 0.102 | 0.109 | 0.158 | 0.134 |
| **NH** | 0.125 | 0.067 | 0.079 | -0.083 | 0.121 | 0.165 | 0.173 | 0.119 | 0.119 | 0.177 | 0.147 |
| **FL** | 0.136 | 0.138 | 0.110 | 0.121 | -0.014 | 0.157 | 0.165 | 0.114 | 0.112 | 0.157 | 0.129 |
| **FM** | 0.153 | 0.170 | 0.144 | 0.165 | 0.157 | -0.012 | 0.187 | 0.143 | 0.140 | 0.183 | 0.159 |
| **MS** | 0.173 | 0.168 | 0.148 | 0.173 | 0.165 | 0.187 | -0.018 | 0.089 | 0.124 | 0.170 | 0.152 |
| **KH** | 0.127 | 0.120 | 0.102 | 0.119 | 0.114 | 0.143 | 0.089 | 0.001 | 0.070 | 0.120 | 0.105 |
| **VH** | 0.130 | 0.127 | 0.109 | 0.119 | 0.112 | 0.140 | 0.124 | 0.070 | 0.001 | 0.088 | 0.090 |
| **SH** | 0.177 | 0.186 | 0.158 | 0.177 | 0.157 | 0.183 | 0.170 | 0.120 | 0.088 | -0.007 | 0.121 |
| **DH** | 0.158 | 0.159 | 0.134 | 0.147 | 0.129 | 0.159 | 0.152 | 0.105 | 0.090 | 0.121 | 0.009 |


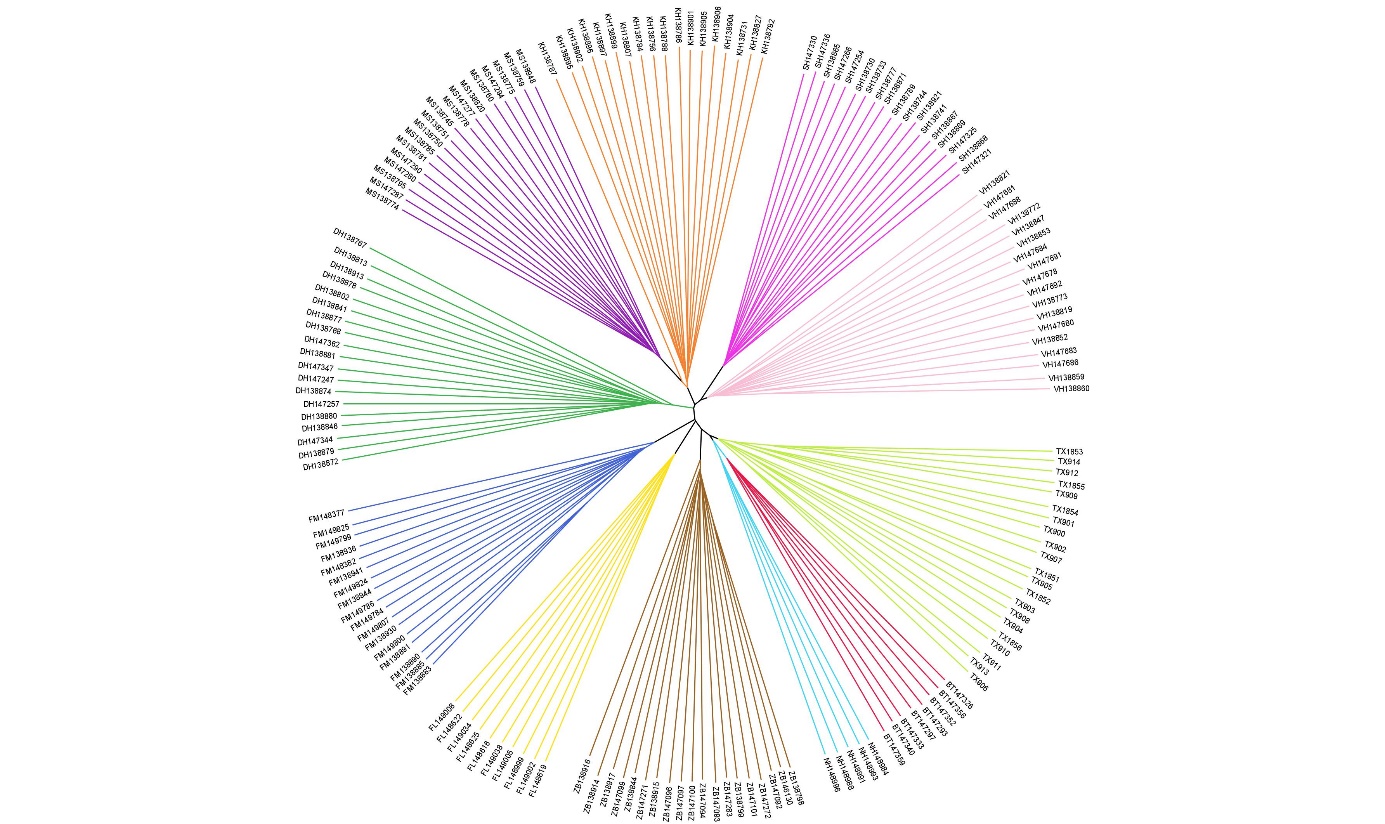


*Supplemental* *Figure 2: The Neighbour Joining Tree of all individual sheep. Different breeds are indicated with different colors. Dark purple is Mergelland, orange is Kempen heath sheep, light purple is Schoonebeeker heath sheep, pink is Veluwe heath sheep, lime green is North Hollander, red is Blue Texel, light blue is Texel, brown is Zwartbles, yellow is Flevolander, blue is Friesian milk sheep and green is Drenthe heath sheep.*
